# Genome-Wide Gene Expression Profiles in Lung Tissues of Pig Breeds Differing in Resistance to Porcine Reproductive and Respiratory Syndrome Virus

**DOI:** 10.1371/journal.pone.0086101

**Published:** 2014-01-23

**Authors:** Jinyi Xing, Feng Xing, Chenhua Zhang, Yujie Zhang, Nan Wang, Yanping Li, Lijuan Yang, Chenglan Jiang, Chaoyang Zhang, Changhong Wen, Yunliang Jiang

**Affiliations:** 1 College of Animal Science and Veterinary Medicine, Shandong Agricultural University, Tai’an, Shandong Province, The People’s Republic of China; 2 College of Life Science, Linyi University, Linyi, Shandong Province, The People’s Republic of China; 3 College of Animal Science, Tarim University, Ala'er, Xinjiang Uygur Autonomous Region, The People’s Republic of China; 4 Department of Mathematics, The University of Southern Mississippi, Hattiesburg, Mississippi, United States of America; 5 School of Computing, The University of Southern Mississippi, Hattiesburg, Mississippi, United States of America; Huazhong Agricultural University, China

## Abstract

Porcine reproductive and respiratory syndrome (PRRS) caused by PRRS virus (PRRSV) is an infectious disease characterized by severe reproductive deficiency in pregnant sows, typical respiratory symptoms in piglets, and high mortality rate of piglets. In this study, we employed an Affymetrix microarray chip to compare the gene expression profiles of lung tissue samples from Dapulian (DPL) pigs (a Chinese indigenous pig breed) and Duroc×Landrace×Yorkshire (DLY) pigs after infection with PRRSV. During infection with PRRSV, the DLY pigs exhibited a range of clinical features that typify the disease, whereas the DPL pigs showed only mild signs of the disease. Overall, the DPL group had a lower percentage of CD4^+^ cells and lower CD4^+^/CD8^+^ratios than the DLY group (*p*<0.05). For both IL-10 and TNF-α, the DLY pigs had significantly higher levels than the DPL pigs (*p*<0.01). The DLY pigs have lower serum IFN-γ levels than the DPL pigs (*p*<0.01). The serum IgG levels increased slightly from 0 dpi to 7 dpi, and peaked at 14 dpi (*p*<0.0001). Microarray data analysis revealed 16 differentially expressed (DE) genes in the lung tissue samples from the DLY and DPL pigs (q≤5%), of which LOC100516029 and LOC100523005 were up-regulated in the PRRSV-infected DPL pigs, while the other 14 genes were down-regulated in the PRRSV-infected DPL pigs compared with the PRRSV-infected DLY pigs. The mRNA expression levels of 10 out of the 16 DE genes were validated by real-time quantitative RT-PCR and their fold change was consistent with the result of microarray data analysis. We further analyzed the mRNA expression level of 8 differentially expressed genes between the DPL and DLY pigs for both uninfected and infected groups, and found that TF and USP18 genes were important in underlying porcine resistance or susceptibility to PRRSV.

## Introduction

Porcine reproductive and respiratory syndrome (PRRS), also known as blue-ear pig disease, is a widespread infectious disease caused by porcine reproductive and respiratory syndrome virus (PRRSV), which is characterized by severe reproductive deficiency in pregnant sows, typical respiratory symptoms in pigs of all ages, increased susceptibility to secondary bacterial infection, and high mortality rate of piglets [1], [2], [3]. It was first reported in 1987 in North America and Central Europe, and is now one of the most severe diseases in the swine industry, costing approximately $560 million each year in the United States alone [4], [5]. The PRRSV is a member of the enveloped, positive-sense, single-stranded RNA virus family *Arteriviridae* [6] and exhibits a very narrow host cell tropism. PRRSV-infected pigs are susceptible to pneumonia and growth reduction, while infected sows have increased rates of abortion, stillbirth, and mummification, and also give birth to weak piglets with chronic respiratory problems [7].

Breed is one of the factors that determines resistance or susceptibility to PRRSV in pigs [8]. Experimental PRRSV challenge infections in Duroc, Hampshire and Meishan pigs revealed that the latter breed had significantly less PRRSV antigen in their lungs than the other breeds, suggesting that differences in the severity and distribution of PRRSV-induced lesions and normal serum antibody responses to PRRSV may be influenced, at least in part, by genetic factors [9]. Another two independent studies indicated that different pig breeds or lines responded differently to PRRSV infection, while analysis of the serum cytokine levels confirmed gene expression differences in the spleens and bronchial lymph nodes of these pigs [10], [11]. Our previous study revealed differences in the PRRSV copy number and NMMHC-IIA and CD163 mRNA expression levels between virus infected Dapulian (DPL) and Duroc×Landrace×Yorkshire (DLY) pigs [12].

Since the early 1990s, cDNA microarray genomic technology has been used to simultaneously assess mRNA transcription patterns for thousands of genes simultaneously, and has been commonly employed for determining patterns of differential gene expression in a wide range of organisms, thereby attracting considerable attention from the biomedical research community [13], [14]. Microarrays are particularly useful for studying whether cellular mRNAs, differentially regulated by each viral protein, play a crucial role for virus multiplication in the cell and interactions between the host and virus [15], [16]. Several microarray studies have reported that many genes are involved in porcine responses to infection with PRRSV [16], [17], [18].

DPL pig, a breed of indigenous Chinese pigs which is native in Jining City, Shandong Province, China, has some desirable traits, such as strong disease-resistance, high prolificacy and good meat quality. During the summer of 2006, a large-scale devastating outbreak of PRRS overwhelmed about 10 provinces in China, resulting in more than 2 million infected pigs and a death toll of at least 0.4 million animals [19]. However, the DPL pigs showed strong resistance to PRRSV infection and only a few individuals died. The objective of this study was to examine the gene expression differences between lung tissues from DPL and DLY pigs infected with PRRSV (strain JXA1), a highly pathogenic virus isolated from DLY commercial pig stocks, to identify genes associated with PRRSV-resistance. To achieve this objective, we used Affymetrix microarray technology to examine gene expression profiles in the PRRSV infected DPL- and DLY- pigs.

## Materials and Methods

### Animals and Tissue Collection

All animal procedures were performed according to protocols approved by the Animal Care and Use Committee of Shandong Agricultural University. Sixteen healthy 30-day-old weaned DPL pigs were selected from Jiaxiang Dapulian Farm, Jining City, China, and 15 healthy 30-day-old weaned DLY pigs were obtained from a commercial farm with high standards of animal health. These pigs were free from PRRSV, porcine circovirus type 2 (PCV2), pseudorabies virus (PRV), and classical swine fever virus (CSFV) as determined by ELISA tests for serum antibodies; the absence of PRRSV was also confirmed by real-time quantitative reverse transcription PCR (qRT-PCR). Pigs were randomly assigned into two groups and reared in separate places: the PRRSV-infected group consisted of eleven DPL and ten DLY pigs, and the control group consisted of five DPL and five DLY pigs. Infection in the pigs was administrated via inoculation with 2 ml of a viral suspension of PRRSV (at a tissue culture infectious dose of 10^5^) by dripping the solution into the nasal cavity of each pig. The control group was treated with an identical volume of phosphate buffer saline (PBS) by the same method. Rectal temperatures and clinical examinations on the pigs were recorded daily during the experiment. Anticoagulant-treated blood and untreated blood samples were collected separately at 0, 7, 14, and 21 days post-infection (dpi) from the infected and control groups for assaying CD4^+^, CD8^+^, cytokines (interleukin (IL) 1 beta (IL-1β), IL-2, IL-10), interferon (IFN)-gamma (IFN-γ), tumor necrosis factor-alpha (TNF-α), and immunoglobulin G (IgG) protein levels. Lung samples for microarray analysis and real-time qRT-PCR analysis were collected from six infected DLY and DPL pigs (three pigs for each breed) immediately post-slaughter at 28 dpi.

### Flow Cytometry and ELISA Assays

Peripheral blood mononuclear cells (PBMC) were separated from whole blood at 0, 7, 14 and 21 dpi by density-gradient centrifugation with Histopaque 1077 (Sigma, Steinheim, Germany). Phenotypic analysis of the PBMC subsets was done by flow cytometry using anti-CD4-FITC and anti-CD8-RPE monoclonal antibodies (AbD Serotec, Kidlington, UK). Analyses were conducted using an Easy Cyte Mini Cytometer System (Guava, Billerica, MA, USA) at an excitation wavelength of 488 nm. Sera collected from the infected pigs at 0, 7 and 14 dpi were used for cytokine detection (IL-1β, IL-2, IL-10, IFN-γ, TNF-α) and IgG levels by ELISA (R & D Systems, Minneapolis, MN, USA) according to manufacturer’s instructions.

### Microarray Hybridization

Utilizing the porcine Affymetrix GeneChip to compare the mRNA expression profiles of lung tissue samples from the PRRSV-infected DPL pigs with those from DLY pigs was carried out at Capital Biochip Limited Company, Beijing, China. In brief, total RNA was isolated from lung tissue samples and purified using an RNeasy Mini kit (Qiagen, Shanghai, China) according to the manufacturer’s protocol. RNA was prepared using the GeneChip (AFF**-**900623) one cycle target for the labeling and control reagents, and the labeled RNA was hybridized in an Affymetrix Hybridization Oven 640. After hybridization, the microarray slides were washed and dyed with the Affymetrix Fluidics Station 450. The probe arrays were scanned with an Affymetrix GeneChip Scanner 3000. The signal intensities of the spots on each image were quantified by the Affymetrix GeneChip Operating Software Version 1.4 (GCOS 1.4), and the data were adjusted and normalized by the dChip software.

### Microarray Data Analysis

The collected microarray data were MIAME compliant. The raw data (tag sequences and counts) have been deposited in the NCBI Gene Expression Omnibus (GEO) with accession number GSE49306.

Microarray data was analyzed with BRB-ArrayTools software developed by Dr. Richard Simon and BRB-Array Tools Development Team and available at http://linus.nci.nih.gov/BRB-ArrayTools.html. The class comparisons for identifying differentially expressed (DE) genes between the PRRSV-infected DPL and DLY pigs were performed with two-sample t-test with *q*-value ≤5% to control false discovery rate and a fold change of 1.5 as a threshold [20]. We also utilized BRB-ArrayTools to obtain hierarchical clustering results and to graphically represent DE genes between the DPL and the DLY PRRSV-infected pigs. Gene ontology (GO) analyses were conducted using the NetAffx (http://www.affymetrix.com) and AgBase (http://www.agbase.msstate.edu/cgi-bin/tools/goslimviewer_select.pl) tools for comparative gene ontology categories, including molecular function (MF), biological process (BP) and cellular component (CC). The DE genes were also imported into the online software KEGG (http://www.genome.jp/kegg/pathway.html) for biological pathway mapping.

### Real-time Quantitative Reverse Transcription PCR

Real-time qRT-PCR was employed to validate the microarray data analysis results and to compare mRNA expression levels of DE genes. Genes were selected based on the magnitude of changes in gene expression and its relevance to immune function, the following 10 genes were selected: adenosine monophosphate deaminase 3 (AMPD3), torsinA interacting protein 2 (TOR1AIP2), thymosin beta-15A-like (LOC100525848, THYMOSIN), PRVE (LOC100151993), C6ORF52 (LOC100270682, chromosome 6 open reading frame 52-like), HSPCB (LOC100396742), cytochrome P450 2J2-like (CYP2J2), coiled-coil domain-containing protein 84-like (LOC100516029, CCDC84), ACOX3 (LOC100513192), and the inactive progesterone receptor (PTGES3, LOC100155956). Furthermore, the expression levels of eight more genes AMPD3, ACOX3, CYP3A88, LPL (Lipoprotein lipase), MRC1 (mannose receptor, C type 1), USP18 (ubiquitin sqecific peptidase 18), TF (transferrin) and ENPEP (glutamyl aminopeptidase (aminopeptidase A)), were compared in both uninfected and infected DPL pigs with those in the uninfected and infected DLY pigs. The RNA samples prepared for microarray analysis were also used for real-time qRT-PCR validation. cDNA synthesis was conducted according to the manufacturer’s instruction and the primer sequences for these reactions are shown in Table 1. qRT-PCR was carried out with the Brilliant SYBR Green qRT-PCR Master Mix (Stratagene, La Jolla, CA, USA). All PCR reactions were performed in triplicate with negative controls. Using the GAPDH gene for normalization, the relative expression levels of the DE genes were analyzed by the 2^−ΔΔCT^ method [21].

**Table 1:** Primers used for qRT-PCR.

Gene	Sequence (5'→3')	Ampliconsize (bp)	Annealingtemperature (°C)	GenBank accessionnumber
CCDC84	TCACAGCAGTCCTCATACTTG	185	57	XM_003129917
	GGTCCTATTTGTTGGCTTCC			
THYMOSIN	GAAGTGGAGAAGTTTGACAGG	87	57	XM_003135273
	CTGCTGGATAGTTTCCTTTGAG			
C6ORF52	GGACCCAACCTTTATTCTGC	186	57	NM_001145022
	CGAGTCGTAGAGTTCTTCGC			
PRVE	TTCTCTGGACTGGAATGATG	222	57	XM_001925337.1
	TAGCGTTCTTGTGCTGATG			
HSPCB	TCTCAGTTACCAAGGAGGGC	170	57	XM_001929570
	GCAACAGGGTGAAGACACA			
CYP2J2	AGTTGATACCACCCTGGCT	115	57	XM_003127962
	TGGATTGAATGTGTCTGGG			
AMPD3	GGCTGAGAAGGTGTTCGC	231	57	XM_003353954
	TTCCAATCTTGCTGCGTC			
TOR1AIP2	GAGGCTGAGAGACACCA	295	59	NM_001243389
	GGAGAAACTTCCGTCCT			
ACOX3	ATTCGCCATGTTCCACCAGG	156	58	XM_003134878
	ATAAAGACCCGGCTTGTGG			
PTGES3	TTCTGCAAAGTGGTACGATCGA	187	53	XM_001929413
	GCTTAAAATTATCACTTCCTCCG			
USP18	CAATGACTCCAATGTCTGTT	140	59	NM_213826
	TCTGAAGGTGTAGATGCATC			
CYP3A88	AGGTCTGTCCAATGAAGAACT	152	59	NM_001134824
	AAGGTCGCATCAATCTCCT			
LPL	TTTGAGAAAGGGCTCTGC	191	59	NM_214286
	TGGTTGGTGTGGGTATCAC			
MRC1	GGCTTACGGCGAACCTAATA	139	59	NM_001255969
	TGGTGTTTGTCCTTTGCG			
TF	AGGGATAAAGAAGCAGATGC	134	59	NM_001244653
	GCGTAATAATGGGTTTGT			
ENPEP	GTCTCTACCACCTGACGATG	187	59	NM_214017
	CTGGCATCTGTTGGTTCAT			
GAPDH	ACTCACTCTTCTACCTTTGATGCT	100	57	DQ845173
	TGTTGCTGTAGCCAAATTCA			

### Statistical Analysis

CD4^+^ and CD8^+^ T cell numbers, and their ratios as well as the cytokine and IgG levels in both groups were repeated measures on the same pigs. These data sets are typical longitudinal data sets. We analyzed these data sets with linear mixed-effects model fit by restricted maximum likelihood procedure. This was done with lme function in R package nlme. The significance level of 0.05 was chosen for testing the difference between different pig breeds. Two-sample t-test and Wilcoxon rank test were employed to compare the mRNA expression levels of DE genes.

## Results

### Clinical Features of PRRSV-infected Pigs

The PRRSV-infected DLY pigs showed the following clinical symptoms within 3 days of infection: anorexia, depression, diarrhea, reluctance to drink, and wasting. At 14 dpi, most of the PRRSV-infected DLY pigs had signs of coughing, sneezing, conjunctivitis, eye discharge, increased respiratory rates, and constipation. Some DLY pigs exhibited shivering, spasms, cyanosis, and reddening of the ear skin after 20 dpi. On the contrary, the PRRSV-infected DPL pigs exhibited only mild depression and did not show any obvious clinical characteristics of the disease. The rectal temperatures of the DLY pigs revealed a persistent high fever (above 39.6°C) after 18 dpi, which was higher than in the DPL pigs.

### CD4^+^ and CD8^+^ T lymphocytes

The percentage of CD4^+^ cells in the peripheral blood samples from both DPL and DLY pigs significantly increased from 0 dpi to 7 dpi (*p*<0.05), then decreased to lower levels at 14 and 21 dpi, which were not significantly different with that at 0 dpi (*p*>0.05) (Table S1). Overall, the DPL group had a lower percentage of CD4^+^ cells than the DLY group (*p*<0.05). In contrast, the percentage of CD8^+^ T cells in peripheral blood samples from both DPL and DLY pigs did not show significant difference (*p*>0.05); and it was significantly lower at 7 and 21 dpi than at 0 and 14 dpi (*p*<0.05) (Table S1). Furthermore, the DPL pigs had relatively lower CD4^+^/CD8^+^ratios than the DLY pigs (*p*<0.05), the CD4^+^/CD8^+^ratio at 0, 7 and 21 dpi did not show significant difference (*p*>0.05) and was significantly lower than that at 14 dpi (*p*<0.01) (Table S1).

### Cytokines and IgG

During 0–14 days after PRRSV infection, the change of IL-1β and IL-2 was not significantly different between the DPL and the DLY pigs (*p*>0.05) although both IL-1β and IL-2 levels were much higher at 7 dpi than those at 0 (*p*<0.01) and 14 dpi (Table S2). For both IL-10 and TNF-α, the DLY pigs had significantly higher levels than the DPL pigs (*p*<0.01); for both the DPL and the DLY pigs, their IL-10 and TNF-α levels increased from 0 dpi to 7 dpi (*p*<0.001), decreased from 7 dpi to 14 dpi, at which the levels were significantly lower than those at 0 dpi (*p*<0.01) (Table S2). In contrast, serum IFN-γ levels fell from 0 dpi to 7 dpi (*p*<0.01), then increased at 14 dpi; significant differences between DPL and DLY pigs were detected across the whole experiment (*p*<0.01), more specifically, the DLY pigs have lower serum IFN-γ levels than the DPL pigs (*p*<0.01) (Table S2). Overall, the serum IgG levels increased slightly from 0 dpi to 7 dpi, which is not statistically significant (*p*>0.05), and peaked at 14 dpi (*p*<0.0001) (Table S2). In addition, the PRRSV-infected DLY pigs had slightly higher IgG levels than the PRRSV-infected DPL pigs during 0–14 dpi (Table S2); however, no statistically significant differences were detected between the DPL and the DLY pigs (*p*>0.05).

### Microarray Gene Expression Analysis

**Figure 1:**
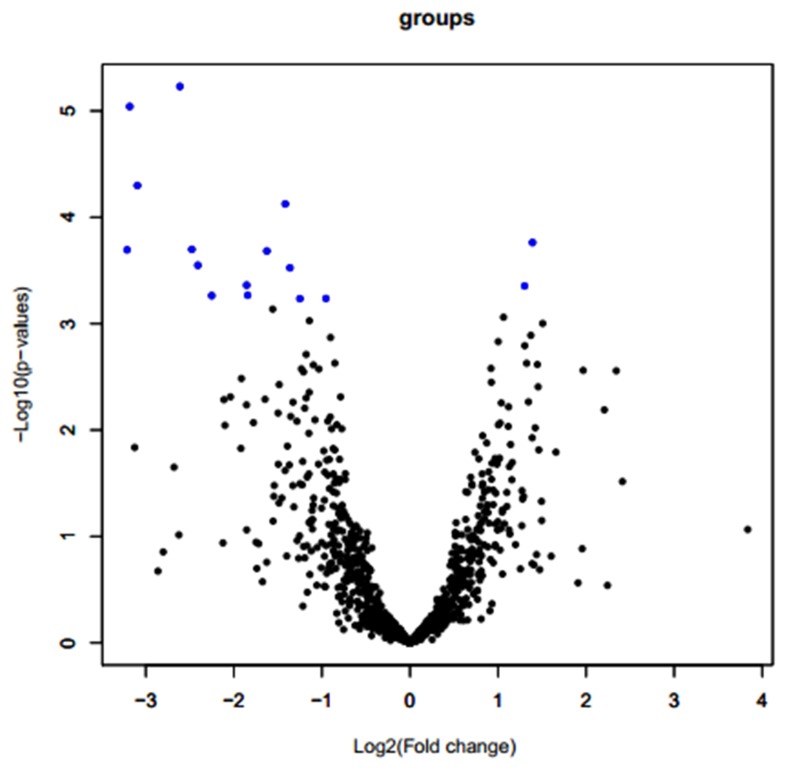
Volcano plots depicting estimated fold changes (log2, x-axis) and statistically significant differences (−log10, *p*-value, y-axis). Each point represents a gene, and colors correspond to the ranges of the negative log_10_ *P* and log_2_ fold change values. Blue circles: differentially expressed genes. Fold changes >0 indicate up-regulated genes, whereas fold changes <0 indicate down-regulated genes. Black circles: no statistically significant expressed genes.

Lung samples collected from six of the PRRSV-infected pigs (three DPL and three DLY) at 28 dpi were analyzed by using a porcine Affymetrix GeneChip. First, the gene probe sets were filtered to eliminate those with very low expression summary values and low variability across all of the samples. The remaining 1419 transcripts were used for subsequent analysis (Table S3). After quantile normalization, 16 genes showed differential expression at the *q*≤5% level (Table 2, Figure 1), among which two genes (LOC100516029 and LOC100523005) were up-regulated by a fold change of 2.63 and 2.47 in the infected DPL pigs compared to those in the infected DLY pigs, respectively. The other fourteen genes were down-regulated in the infected DPL pigs compared with the infected DLY pigs.

**Table 2:** Differentially expressed genes between PRRSV-infected DPL and DLY pigs.

Probe Set ID	Gene Symbol(Gene ID)	Gene Title	FoldChange	Parametricp-value	Description
Ssc.1008.1.A1_at	–	clone: LVR010042C12, expressedin liver	0.18	0.0002005	liver
Ssc.12855.1.S1_at	–	from clone CH242-486P11 onchromosome X, expressed inpituitary gland	0.12	5.03e-05	pituitary gland
Ssc.1527.2.A1_at	–	–	0.28	0.0005407	–
Ssc.15638.1.A1_at	–	*Sus scrofa* adenosine monophosphatedeaminase 3 (AMPD3)	0.39	0.000299	–
Ssc.1620.1.A1_at	–	*Sus scrofa* torsinA interacting protein 2(TOR1AIP2)	0.38	7.5e-05	–
Ssc.17284.1.A1_at	–	–	0.11	9.1e-06	–
Ssc.18841.1.A1_at	–	–	0.52	0.0005808	–
Ssc.25850.1.A1_at	LOC100155956	inactive progesterone receptor,23 kDa (PTGES3)	0.21	0.0005463	inactive progesterone receptor
Ssc.26266.1.S1_at	LOC100525848	thymosin beta-15A-like (THYMOSIN)	0.11	0.0002024	thymosin beta-15A-like
Ssc.26819.1.A1_at	LOC100151993	similar to FYVE, RhoGEF and PHdomain-containing protein 4 (Actinfilament-binding protein frabin)(FGD1-related F-actin-binding protein)(Zinc finger FYVE domain-containingprotein 6) (PRVE)	0.42	0.0005825	alveolar macrophage
Ssc.28462.1.A1_a_at	LOC100270682	chromosome 6 open reading frame52-like (C6ORF52)	0.28	0.0004352	chromosome 6 open reading frame 52-like
Ssc.29634.1.A1_at	HSPCB LOC100396742	Heat shock 90 kD protein 1,beta (HSPCB)	0.32	0.0002076	–
Ssc.3880.1.S1_at	LOC100513192	hypothetical protein (ACOX3)	0.16	5.9e-06	hypothetical protein
Ssc.5327.2.A1_at	–	cytochrome P450 2J2-like,expressedin trachea (CYP2J2)	0.19	0.000283	–
Ssc.6964.1.A1_at	LOC100516029	coiled-coil domain-containingprotein 84-like (CCDC84)	2.63	0.000173	spleen
Ssc.7981.1.A1_at	LOC100523005	hypothetical protein	2.47	0.0004426	hypothetical protein

### Cluster Analyses of DE Genes

**Figure 2:**
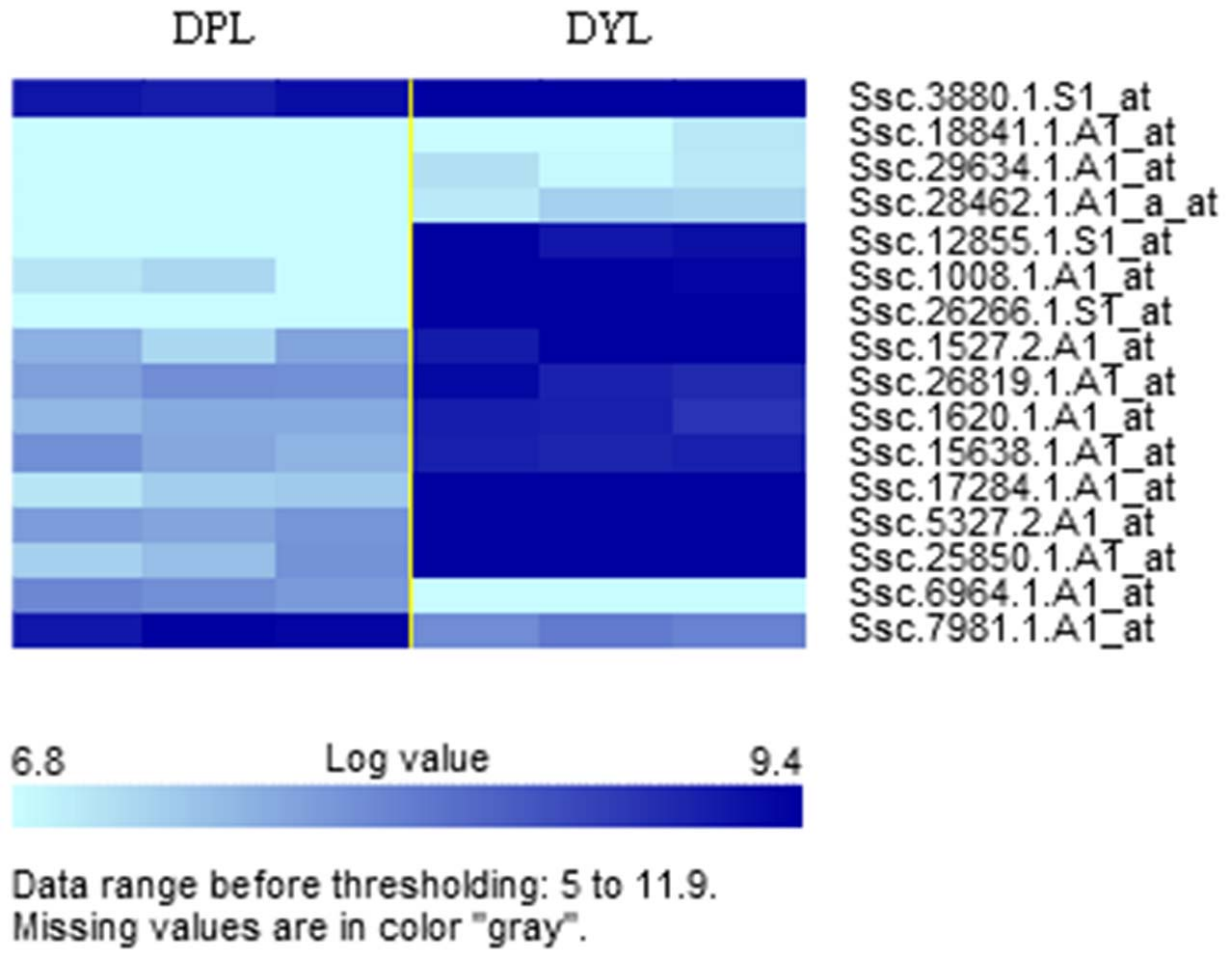
Clustered heat map of the statistically significant expressed genes identified between DPL- and DLY-infected pigs. Each column represents one pig, and each horizontal line refers to one gene. The three columns on the left and right represent the DPL and the DLY pigs, respectively. The color bar at the bottom of the figure indicates the expression level of the genes, those in the lightest blue have lower expression relative to the geometrical means, while dark blue indicates genes with higher expression relative to the geometrical means.

Clustering was used to define sets of genes with similar responses to viral infection at the *q*≤0.005 level. Clustering analysis of gene expression profiles for DPL versus DLY pigs post-infection showed that most of the 16 genes from the DPL pigs had lower expression levels when compared with those from the DLY pigs (Figure 2).

### GO Annotation of DE Genes

GO analysis revealed 39 classes of genes, many of which share common transcripts and are grouped into 20 BP, 13 MF and 6 CC (Table 3, Table S4). Most genes in the BP class are related to metabolic processes, but catalytic activity and binding are only represented in the MF group.

**Table 3:** Gene ontology (GO) analysis of differentially expressed genes.

GO category	GO term	GO description	Number of genes
GO: 0008152	BP	metabolic process	13
GO: 0050789	BP	regulation of biological process	5
GO: 0006139	BP	nucleobase-containing compound metabolic process	4
GO: 0006351	BP	transcription, DNA-dependent	4
GO: 0006412	BP	translation	4
GO: 0007165	BP	signal transduction	4
GO: 0007010	BP	cytoskeleton organization	3
GO: 0009058	BP	biosynthetic process	3
GO: 0044238	BP	primary metabolic process	3
GO: 0006520	BP	cellular amino acid metabolic process	2
GO: 0006810	BP	transport	2
GO: 0005975	BP	carbohydrate metabolic process	1
GO: 0006091	BP	generation of precursor metabolites and energy	1
GO: 0006259	BP	DNA metabolic process	1
GO: 0006464	BP	protein modification process	1
GO: 0006811	BP	ion transport	1
GO: 0006996	BP	organelle organization	1
GO: 0009056	BP	catabolic process	1
GO: 0016043	BP	cellular component organization	1
GO: 0019538	BP	protein metabolic process	1
GO: 0003824	MF	catalytic activity	9
GO: 0005488	MF	binding	9
GO: 0004871	MF	signal transducer activity	6
GO: 0004872	MF	receptor activity	6
GO: 0000166	MF	nucleotide binding	5
GO: 0003677	MF	DNA binding	4
GO: 0016740	MF	transferase activity	4
GO: 0004672	MF	protein kinase activity	2
GO: 0003700	MF	sequence-specific DNA binding transcription factor activity	1
GO: 0003779	MF	actin binding	1
GO: 0004518	MF	nuclease activity	1
GO: 0016301	MF	kinase activity	1
GO: 0016787	MF	hydrolase activity	1
GO: 0005575	CC	cellular component	5
GO: 0005623	CC	cell	5
GO: 0005634	CC	nucleus	3
GO: 0005737	CC	cytoplasm	2
GO: 0005856	CC	cytoskeleton	1
GO: 0005886	CC	plasma membrane	1

### Pathway Analysis

Pathway analysis of the DE genes was performed using the KEGG database (http://www.genome.jp/kegg/pathway.html). The results revealed eight main pathway categories (Figure S1): ERBB signaling, endocytosis, JAK-STAT signaling, T cell receptor signaling, regulation of actin cytoskeleton, insulin signaling, bacterial invasion of epithelial cells and chronic myeloid leukemia.

### Validation of the Microarray Data by qRT-PCR

**Figure 3:**
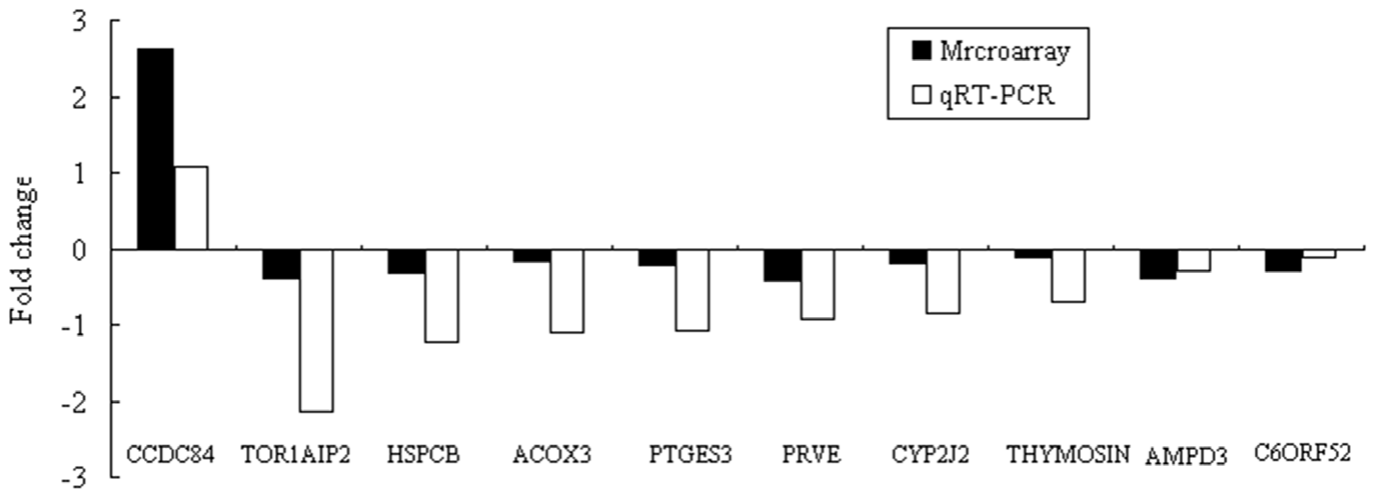
Validation of the 10 differentially expressed genes obtained from microarray analysis by qRT-PCR, n = 3. The *p*-values of the qRT-PCR data are as follows: 0.0067 (C6ORF52), 0.3920 (CCDC84), 0.3661 (THYMOSIN), 0.8195 (PRVE), 0.3154 (HSPCB), 0.6534 (CYP2J2), 0.002 (AMPD3), 0.1622 (TOR1AIP2), 0.8329 (PTGES3), 0.8808 (ACOX3).

We selected 10 of the DE genes identified by microarray analysis for real-time qRT-PCR analysis, including one up-regulated gene (CCDC84, which encodes coiled-coil domain-containing 84) and nine down-regulated genes (C6ORF52, THYMOSIN, PRVE, HSPCB, CYP2J2, AMPD3, TOR1AIP2, PTGES3, ACOX3). The fold change in the mRNA expression levels of the 10 genes between the PRRSV-infected DPL and DLY pigs as determined by qRT-PCR was consistent with the microarray results (Figure 3), suggesting that microarray hybridization is a reliable method for genome-wide analysis of expression profiles in the PRRSV-infected DPL and DLY pigs.

### Comparison of DE Gene mRNA Expressions in Uninfected and Infected DPL and DLY Pigs

**Figure 4:**
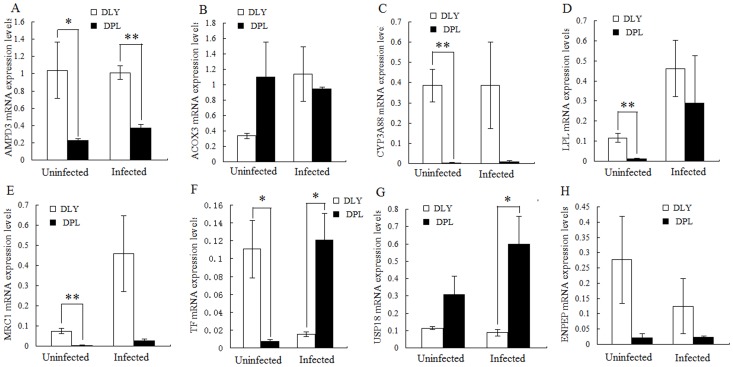
Comparison of 8 gene expression levels for both uninfected and infected DPL pigs with the uninfected and infected DLY pigs. * or ** indicates a statistically significant difference at *p*<0.05 or 0.01, respectively.

In addition, we also analyzed the mRNA expression level of 8 out of 67 genes which are differentially expressed at significance level 0.01 between the DPL and DLY pigs for both uninfected and infected groups. The results showed that AMPD3 gene expression level for both uninfected and infected DPL pigs was significantly lower compared with the uninfected and infected DLY pigs (*p*<0.05, *p*<0.01, Figure 4A). For ACOX3 gene, it did not show significant difference between uninfected DPL and DLY pigs or between infected DPL and DLY pigs (*p*>0.05, Figure 4B). Moreover, three down-regulated genes (CYP3A88, LPL and MRC1) behaved similarly in uninfected pigs, that is, their gene expression levels of uninfected DLY pigs were significantly higher than those of uninfected DPL pigs (*p*<0.01, Figure 4C, D and E), and no significant difference of their mRNA expression levels was detected between infected DLY and DPL pigs (*p*>0.05, Figure 4C, D and E). In uninfected pigs, the mRNA expression level of TF gene was significantly higher in DLY pigs than that in DPL pigs (*p*<0.05, Figure 4F); whereas in infected pigs, it was significantly lower in DLY than that in DPL pigs (*p*<0.05, Figure 4F). USP18 mRNA levels were not significantly different in uninfected groups (*p*>0.05) and were significantly lower in infected DLY pigs than those in infected DPL pigs (*p*<0.05, Figure 4G); ENPEP mRNA expression levels did not show significant difference in uninfected/infected groups of DPL and DLY pigs (*p*>0.05, Figure 4H).

## Discussion

PRRS is pandemic in pigs and causes significant losses to the pig industry via reproductive disorders and growth retardation. In particular, a highly pathogenic disease caused by PRRSV emerged in swine farms all over China from 2006 to 2010, which caused continuous high fever and a high proportion of deaths in vaccinated pigs of all ages [19], [22], [23]. Studies have shown that responses of different pig breeds and lines to PRRSV infection differ greatly [8], [9], [10], [11], [12], [24], [25], [26]. Nevertheless, the molecular mechanisms underlying PRRSV remain largely unknown. In the present study, using Affymetrix GeneChip technology, we have reported the results of a genome-wide comparison of gene expression profiles in lung tissues from two pig breeds differing in resistance to PRRSV. This is the first report of differential expression at the *q*≤5% level for 16 genes that differ between two breeds (Table 2, Figure 1). Observations of interest are the differential responses to PRRSV infection in gene expression, clinical symptoms and biochemical measurements of CD4^+^, CD8^+^, cytokine and IgG profiles between the DPL and the DLY pigs.

The PRRSV-infected DPL and DLY lines showed marked differences in body weight, rectal temperature and serum cytokine levels [10], [11], [27]. Vincent et al. (2006) reported that the serum viral titers at 21 dpi differed among different pig lines after infection with PRRSV [26]. Variation in resistance to PRRSV in Pietrain and Miniature pigs was reported by Reiner et al. [28]. In the present study, we also found differences between DPL and DLY pigs in their clinical features of PRRSV infection: DLY pigs had more severe clinical symptoms and lesions than the DPL pigs, which implied that DPL pigs had a stronger resistance phenotype than DLY pigs when challenged with PRRSV. These results indicate that underlying genetic variation exists in disease susceptibility to PRRSV.

CD4^+^ is a co-receptor that assists the T cell receptor (TCR) to communicate with an antigen-presenting cell [29], while CD8^+^ is a transmembrane glycoprotein that serves as a co-receptor for the TCR [30]. Consequently, CD4^+^ and CD8^+^ T cells play important roles in protection against viral infections. Shimizu et al. observed a remarkable decrease in CD4^+^ T cells after 3 days infection with PRRSV [31]; and this study also reported slight decreases in CD8^+^ T cells at 3 dpi, followed by substantially increased levels [31], while at the same time, the ratios of CD4^+^/CD8^+^ T cells were significantly lower between day 3 and 28 post-inoculation compared with day 0 [31]. A different study revealed that the percentages of CD4^+^ and CD8^+^ T cells were significantly decreased for a few days shortly after PRRSV infection, but returned to pre-infection levels on days 8–10 post-inoculation [32]. Comparing with the results of Shimizu et al. [31] and Nielsen and Bøtner [32], we observed that CD4^+^ T cell numbers increased at 7 dpi, then decreased to its normal range; and the CD8^+^ T cell numbers was lower at 7, 21 dpi than that at 0, 14 dpi. Furthermore, the CD4^+^/CD8^+^ratio was relatively stable from 0 dpi to 21 dpi although it had low value at 14 dpi (Table S1).

Cytokines, including IL-1β, IL-2, IL-8, IL-10, IFN-γ, and TNF-α, have been shown to play important roles in defense against PRRSV [33]. Increased levels of IL-1β, IL-8, IL-10 and TNF-α were present in the porcine PBMC culture supernatant of the PRRSV-infected group as measured by ELISA [34], [35]. Similar findings were reported for IL10, IFN-γ and TNF-α, the levels of which were elevated in the blood of PRRSV-infected pigs [36], [37]. We also observed remarkable increases in IL-1β, IL-2, IL-10 and TNF-α at 7 dpi after which IL-1β and IL-2 dropped to pre-infection levels, IL-10 and TNF-α decreased to lower levels than pre-infection levels. Overall, the IL-10, TNF-α and IFN-γ cytokines in DLY pigs were different with those in DPL pigs (Table S2). They are probably caused by fast PRRSV replication in the DLY pigs and more cytokines production to resist PRRSV replication for DPL pigs, a finding that requires further investigation.

Genome-wide comparisons of the microarray gene expression profiles revealed many complex biologic processes involved in PRRSV infection. By using Affymetrix microarrays, 1409 DE transcripts were identified by analysis of variance, of which 2, 5, 25, 16 and 100 differed between the two breeds by a minimum of 1.5-fold at 1, 3, 6, 9 and 12 h pi, respectively [17]. A study investigating interactions between PRRSV and porcine alveolar macrophages (PAMs) showed that highly pathogenic PRRSV infection affected PAM expression of important genes [18]. The present study identified 16 DE genes using a 1.5-fold change filter, and the number of down-regulated genes (14) was greater than that of the up-regulated genes (2) (Table 2). Cluster analysis of DE genes revealed that most of the 16 genes from the PRRSV-infected DPL pigs had low expression values in comparison with the PRRSV-infected DLY pigs, indicating that they had distinctly different responses to PRRSV infection, thus implying that DPL pigs are more resistant to PRRSV than DLY pigs.

In mice, remote reperfusion lung injury is associated with AMP deaminase 3 activation and attenuation by inosine monophosphate [38], showing that AMPD3 plays a critical role in remote reperfusion lung injury in mice [39]. In this study, the lower AMPD3 expression in DPL pigs compared with their DLY counterparts indicates that the DPL pig lungs were less affected by PRRSV. TorsinA interacting protein 2 (TOR1AIP2), also called LULL1, is a single-pass membrane protein located on the endoplasmic reticulum membrane and nuclear membrane and functions to regulate the distribution of TOR1A between the endoplasmic reticulum and the nuclear envelope (http://www.genecards.org/). Functional defects in the mutant protein are responsible for DYT1 dystonia [40]; however, the role of TOR1AIP2 in lung development is still unknown. Coiled­coil domains typically function in homodimerization and are present in a number of proteins involved in intracellular transport [41]. In this study, we found that expression of the coiled-coil domain-containing protein 84-like (CCDC84) mRNA was 2.63-fold higher in PRRSV-infected DPL pigs than DLY pigs. The role of CCDC84 in resistance to PRRSV requires further study. The other 13 DE genes identified are neither annotated nor encode hypothetical proteins; hence, their functions remain unknown.

DNA microarrays are widely used for measuring the expression levels of large numbers of genes simultaneously; however, microarray results for any given gene are often noisy or ambiguous [42]. Many external factors can significantly affect the accuracy of array data, including RNA preparation, image acquisition, methods for normalization and background subtraction, data processing and standardization, use of visualization tools, and probes used for labeling [43]. At present, real-time qRT-PCR is thought to be the best technique for validating microarray data. However, not all microarray data are verified by this technique. It has been reported that real-time qRT-PCR was able to confirm the change in expression for 17 out of 24 genes identified by high-density filter arrays, and genes showing less than a four-fold difference on the array were not verified by real-time qRT-PCR [44]. A later study revealed that 10 of the genes (10 versus 33) tested that had differential expression in the microarray experiment were not confirmed by the real-time qRT-PCR [45]. Similarly, in our work, 10 out of 16 genes differentially expressed between the PRRSV-infected DPL and DLY pigs as determined by qRT-PCR were consistent with the microarray results. These contradictions between the microarray data and qRT-PCR results probably arise from the greater accuracy of the latter technique compared with microarrays or the lack of specificity in the primer design.

To investigate if the differential expression between the two breeds of infected pigs was confounded with genetic background, 8 genes (6 down-regulated genes and 2 up-regulated genes) were further selected for real-time qRT-PCR analysis in both uninfected and infected groups. The results indicated that the mRNA expression of AMPD3, CYP3A88, LPL, MRC1 and TF genes was significantly different between uninfected DPL and DLY pigs (Figure 4A, C, D, E and F), suggesting that these differential gene expression levels may be also caused by the genetic differences between the two breeds. However, there were genes showed significant changes at expression level between uninfected and infected pigs within breed. In both uninfected and infected groups, the expression differences of AMPD3 between DLY and DPL pigs are similar, suggesting that the difference is not caused by PRRSV infection. The expression of TF gene is significantly lower in DPL pigs than in DLY pigs in uninfected group; however, in infected group, it is much higher in DPL pigs than in DLY pigs (Figure 4F), suggesting that TF gene is likely involved in PRRSV resistance. Similarly, one study indicated that the expression of TF gene was down regulated in high (HR) pigs, with high viremia, low/no weight gain, and many lung lesions [46]. The increase in the mRNA expression in infected DPL pigs compared to uninfected ones suggests a role of TF gene in PRRSV resistance. The expression of CYP3A88, LPL and MRC1 is not significantly different between infected DPL and DLY pigs, indicating that their response to PRRSV infection is not different and they may not control the resistance of DPL pigs to PRRSV infection. The expression of ACOX3 and ENPEP genes were not significantly different between either uninfected or infected DPL and DLY pigs, suggesting that they are not related to PRRSV infection. Studies demonstrated that overexpression of the porcine USP18 leads to reduced replication and/or growth of PRRSV [47] and USP18 restricts PRRSV growth through alteration of nuclear translocation of NF-κB p65 and p50 in MARC-145 cells [48]. In this study, we also noted a slight increase of USP18 mRNA expression level in uninfected DPL pigs compared to uninfected DLY pigs and significantly higher expression level of USP18 gene in infected DPL than that in infected DLY pigs, implying that it also plays an important role in the resistance of DPL pigs to PRRSV infection (Figure 4G).

### Conclusion

In this study, we found that PRRSV-infected DPL pigs showed mild clinical symptoms and had a lower percentage of CD4+ T cells. Moreover, the levels of IL-10, TNF-α and IFN-γ also differed between the PRRSV-infected DPL and DLY pigs. Using Affymetrix microarray chip technology, we compared the gene expression profiles of lung tissues in DPL and DLY pigs after infection with PRRSV and identified sixteen DE genes. We further analyzed the mRNA expression level of 8 out of 67 genes which are differentially expressed at significance level 0.01 between the DPL and DLY pigs for both uninfected and infected groups, and found that TF and USP18 genes were important in underlying porcine resistance or susceptibility to PRRSV.

## Supporting Information

Figure S1 **Signaling pathways of DE genes.** Pathway analysis was mainly based on the KEGG database. A *p*-value of <0.05 and an FDR of <0.05 in the two-side Fisher’s exact test were selected as the statistical significance criteria.
(PDF)

Table S1 **Statistical analysis for the percentage of CD4^+^, CD8^+^ cells and CD4^+^/CD8^+^ ratio.**
(DOC)

Table S2 **Statistical analysis for Cytokines and IgG.**
(DOC)

Table S3 **1419 probe set after filtered by 5% discovery rate and a fold change of 1.5.**
(XLS)

Table S4 **GO annotation of differentially expressed genes.**
(XLS)
