# Supplementary figures and images for: Genome-Wide Gene Expression Profiles in Lung Tissues of Pig Breeds Differing in Resistance to Porcine Reproductive and Respiratory Syndrome Virus
Source: PLoS One. 2014 Jan 23;9(1):e86101. doi: 10.1371/journal.pone.0086101 (PMC3900479; doi:10.1371/journal.pone.0086101)

Ko04012

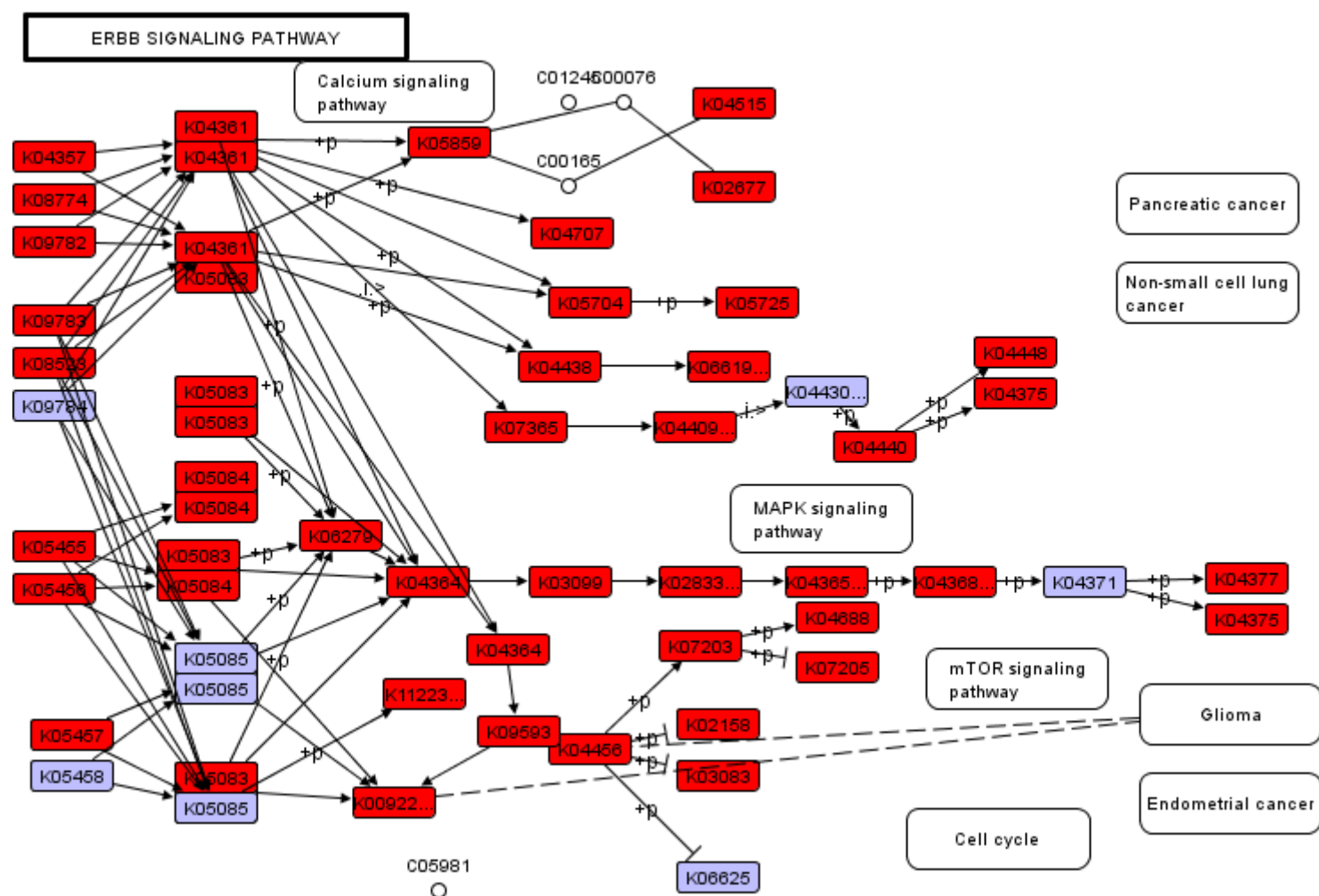

Ko04144

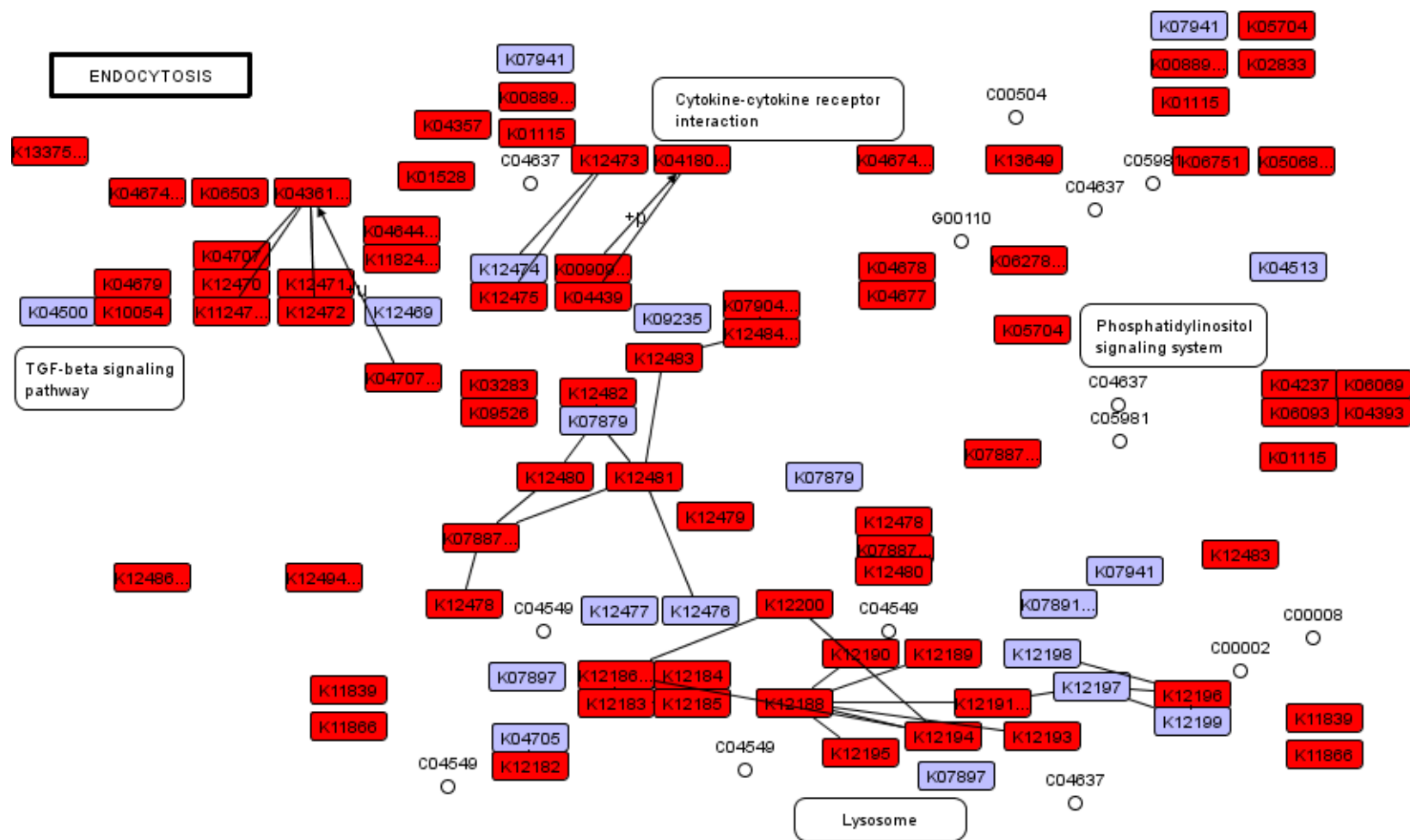

Ko04630

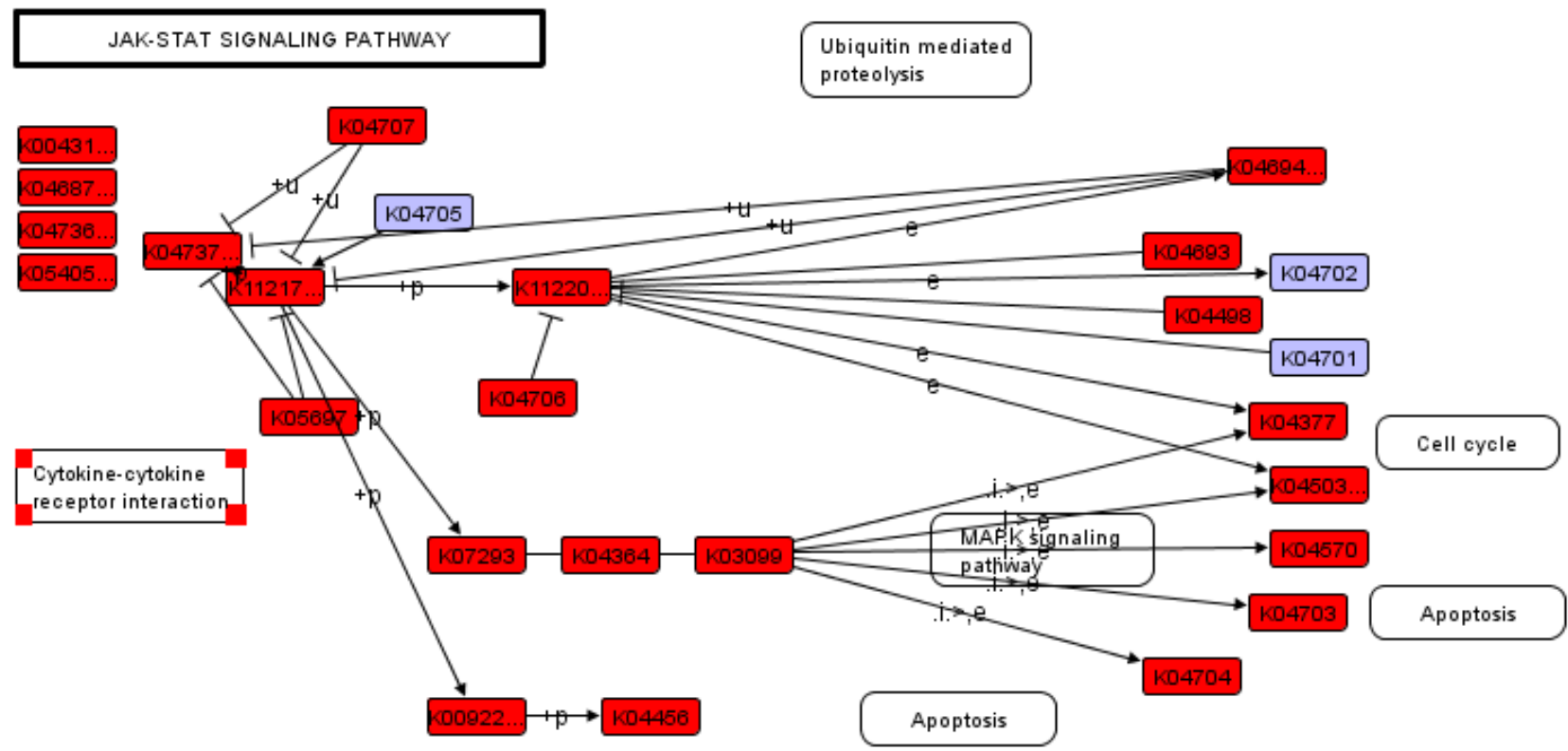

Ko04660

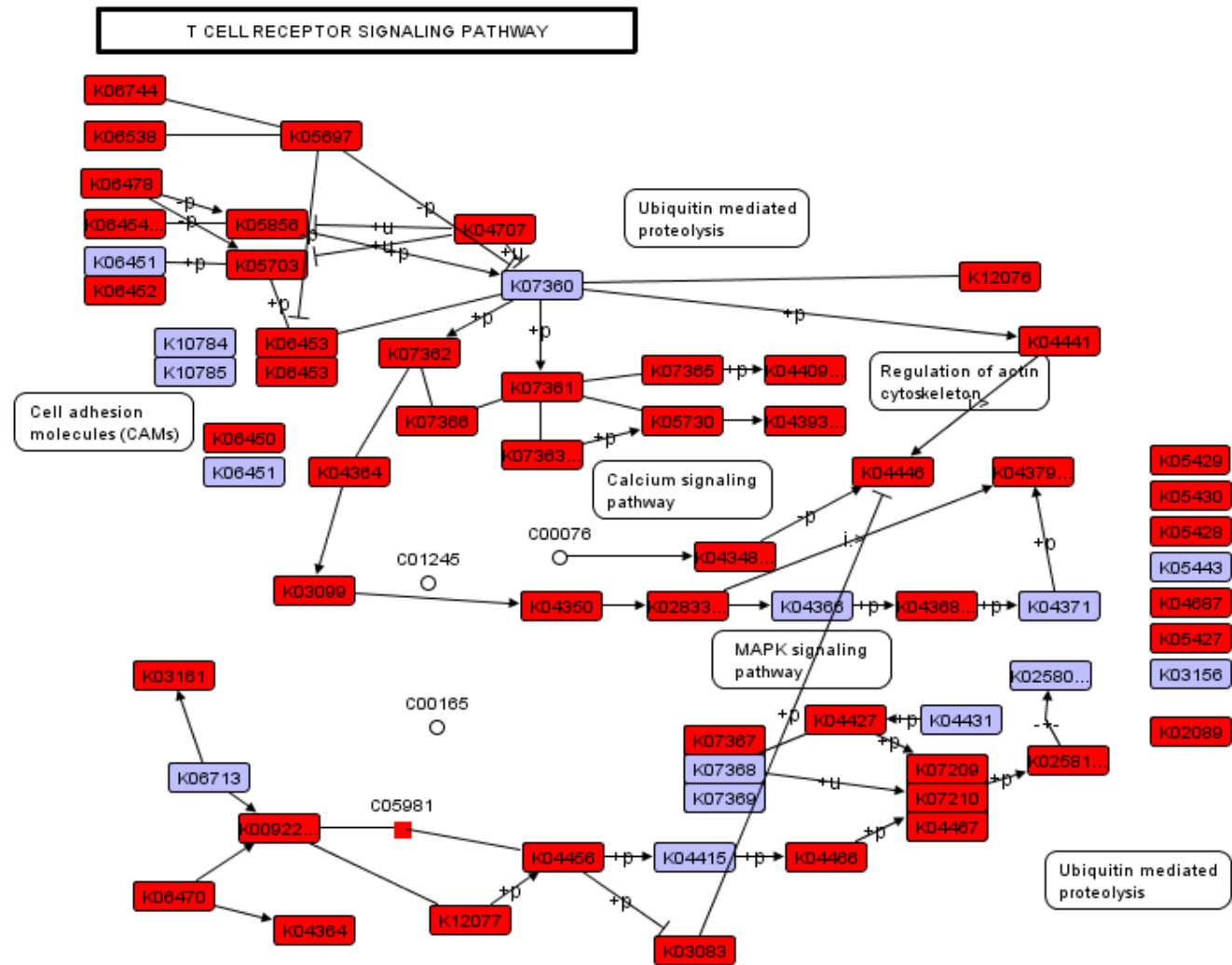

Ko04810

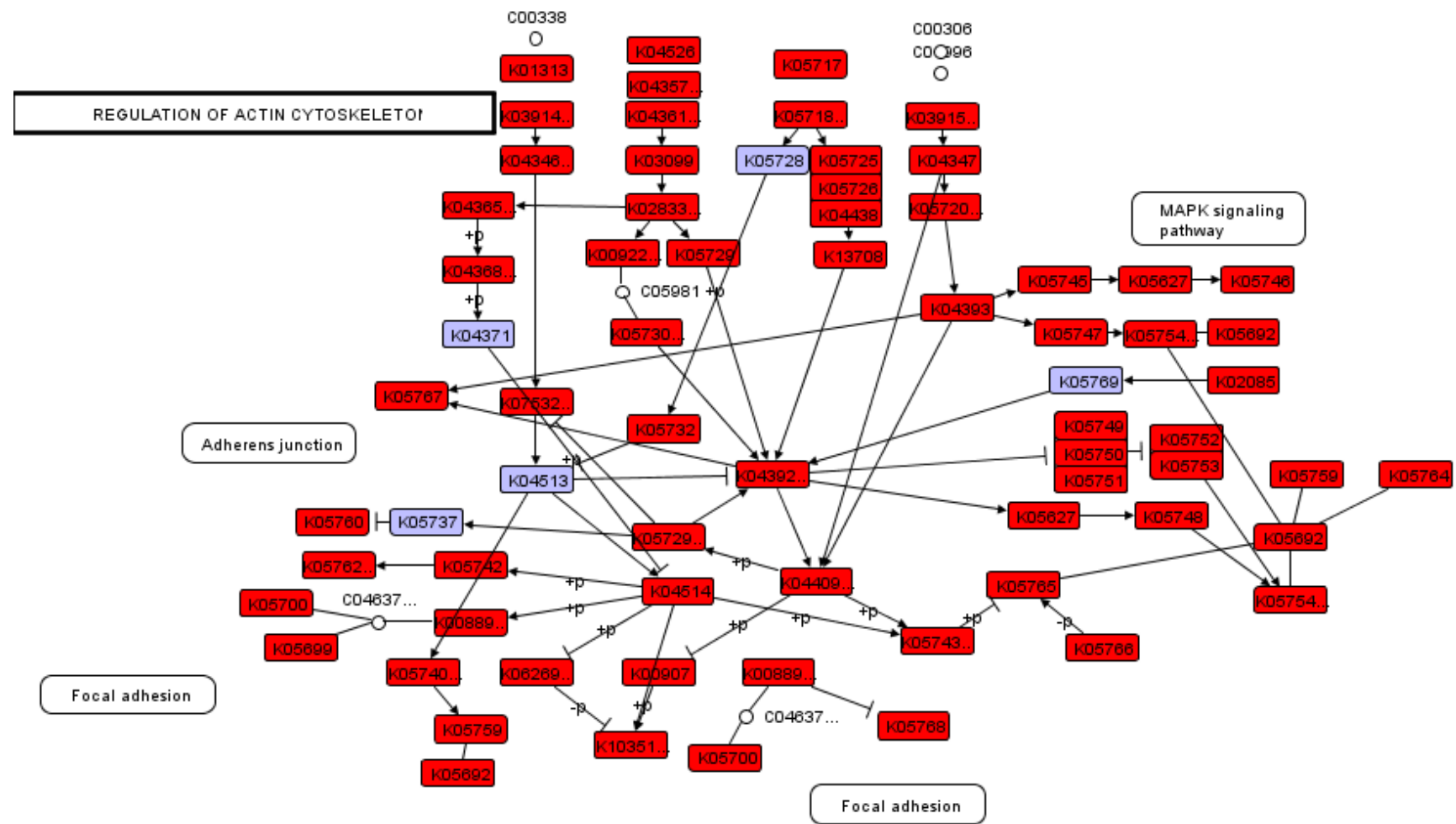

Ko04910

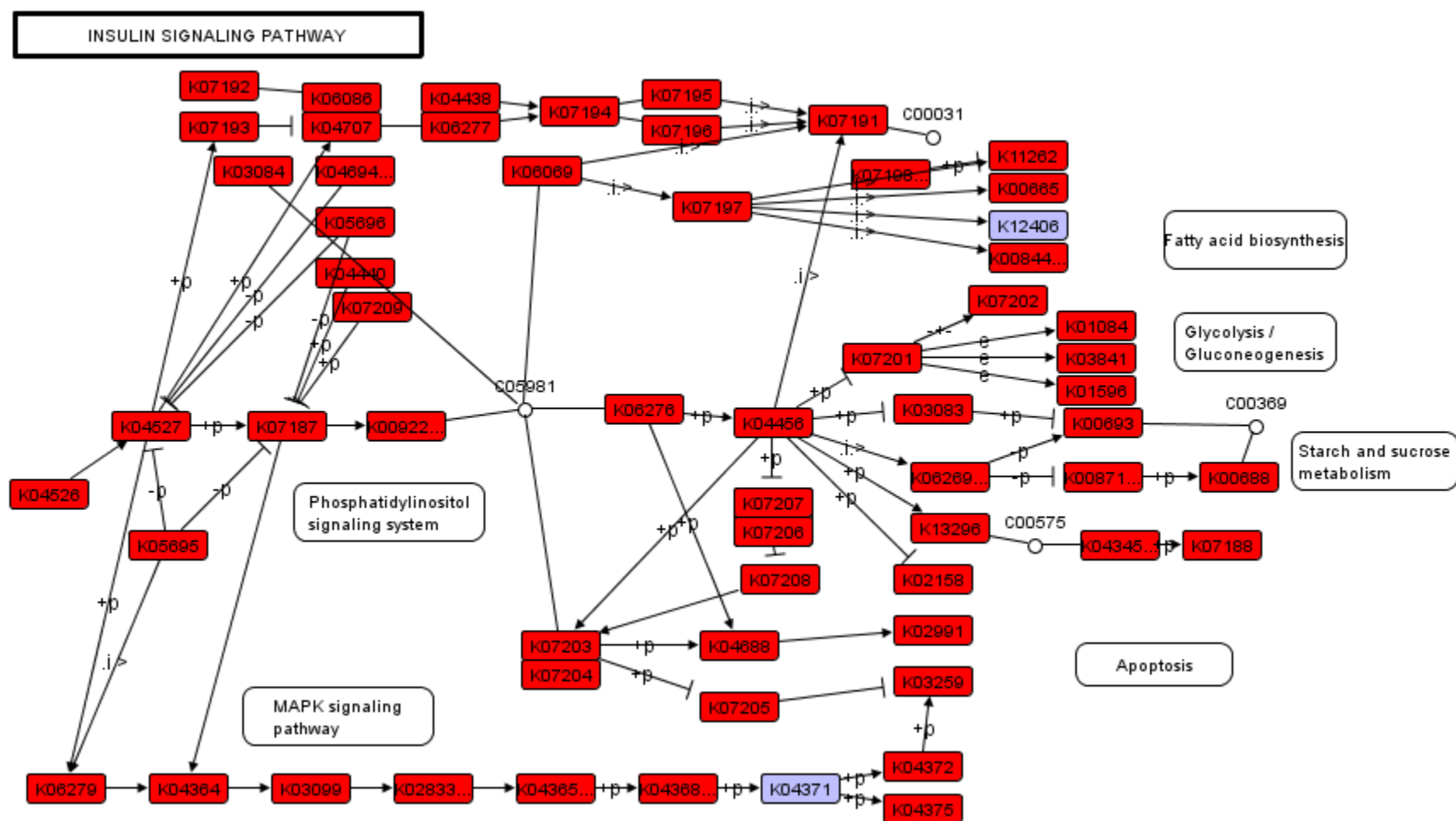

Ko05100

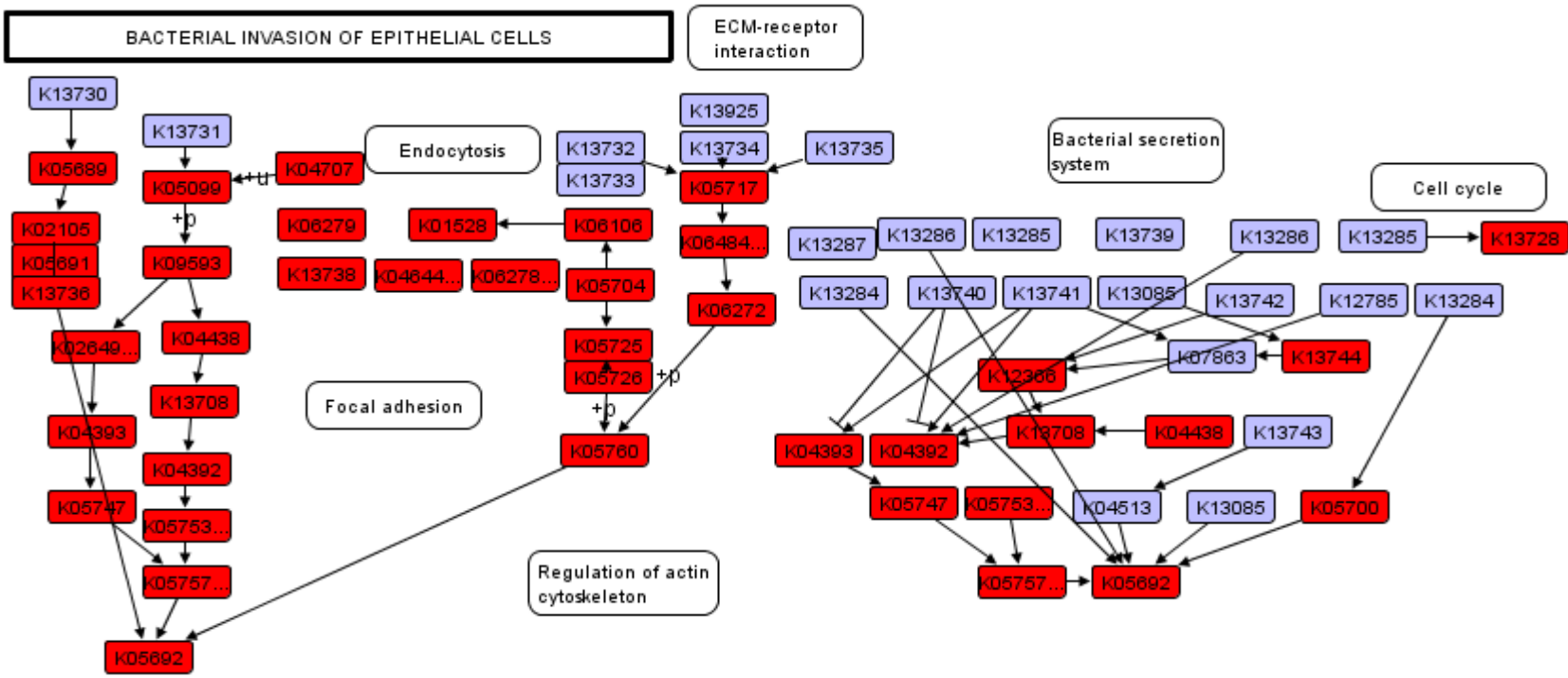

Ko05220

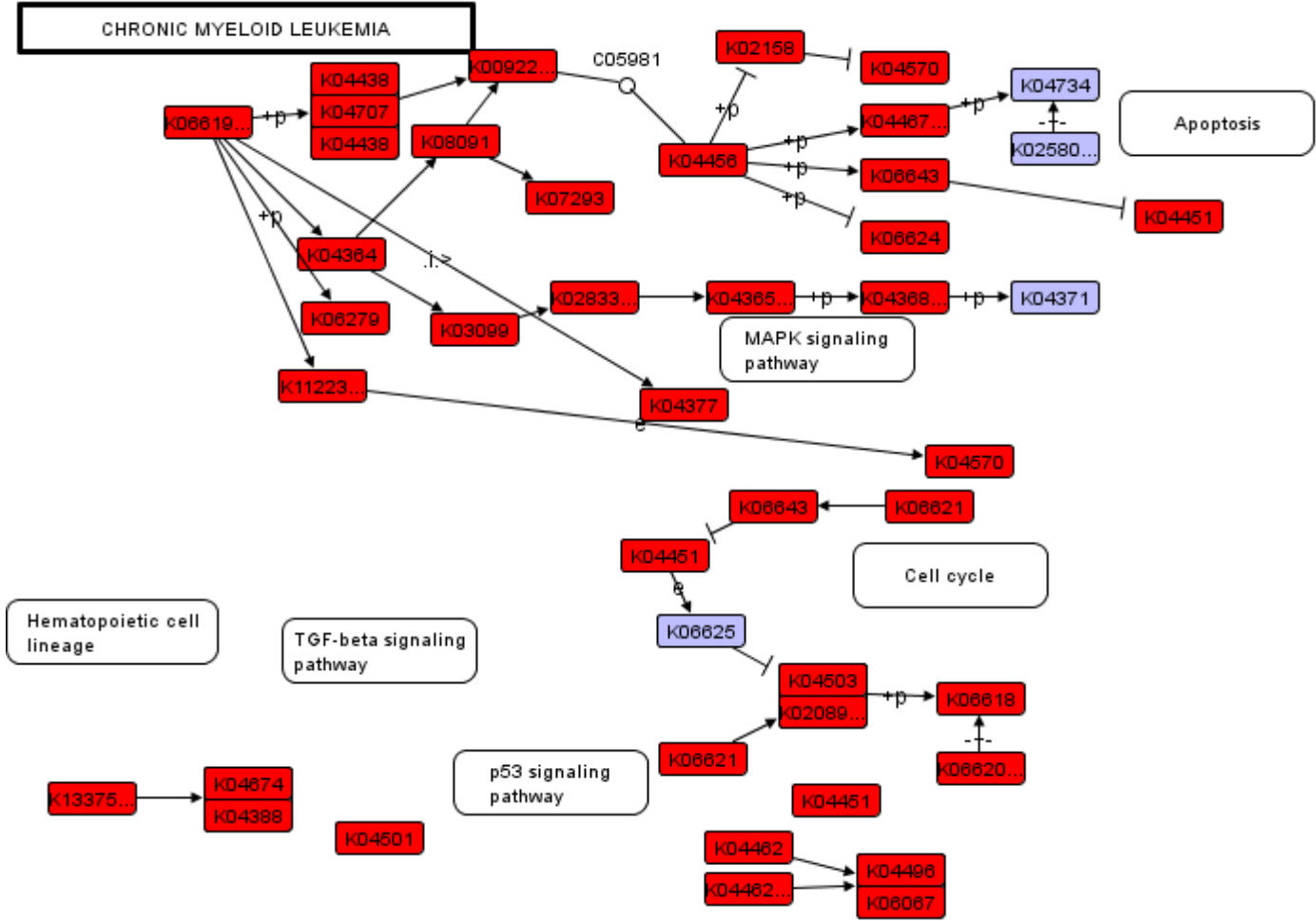

Supplement: Figure S1 — Signaling pathways of DE genes. Pathway analysis was mainly based on the KEGG database. A p-value of <0.05 and an FDR of <0.05 in the two-side Fisher’s exact test were selected as the statistical significance criteria. (PDF) [file pone.0086101.s001.pdf]
