# Supplementary material for: Genome-Wide Gene Expression Profiles in Lung Tissues of Pig Breeds Differing in Resistance to Porcine Reproductive and Respiratory Syndrome Virus
Source: PLoS One. 2014 Jan 23;9(1):e86101. doi: 10.1371/journal.pone.0086101 (PMC3900479; doi:10.1371/journal.pone.0086101)
Supplement: Table S1 — Statistical analysis for the percentage of CD4+, CD8+ cells and CD4+/CD8+ ratio. (DOC) [file pone.0086101.s002.doc]

**Table S1** Statistical analysis for the percentage of CD4^+^, CD8^+^ cells and CD4^+^/CD8^+^ ratio

| Item | Comparison | Difference | Standard error | Degrees of freedom | *t*-value | *p*-value* |
| --- | --- | --- | --- | --- | --- | --- |
| CD4^+^ | DPL - DLY | -4.585000 | 1.310225 | 14 | -2.474448 | 0.0268 |
|  | 7 dpi - 0 dpi | 7.181250 | 2.508890 | 18 | 2.862321 | 0.0104 |
|  | 14 dpi - 0 dpi | -2.118750 | 2.508890 | 18 | -0.844497 | 0.4095 |
|  | 21 dpi - 0 dpi | -1.102083 | 2.508890 | 18 | -0.439271 | 0.6657 |
| CD8^+^ | DPL - DLY | 5.432213 | 4.020112 | 14 | 1.351259 | 0.1980 |
|  | 7 dpi - 0 dpi | -7.695056 | 2.841673 | 21 | -2.707932 | 0.0132 |
|  | 14 dpi - 0 dpi | 3.939944 | 2.841673 | 21 | 1.386487 | 0.1801 |
|  | 21 dpi - 0 dpi | -6.760056 | 2.841673 | 21 | -2.378900 | 0.0269 |
| CD4^+^/CD8^+^ | DPL - DLY | -0.6097838 | 0.216771 | 14 | -2.813031 | 0.0138 |
|  | 7 dpi - 0 dpi | -0.0232860 | 0.201326 | 18 | -0.115663 | 0.9092 |
|  | 14 dpi - 0 dpi | -0.7063077 | 0.201326 | 18 | -3.508271 | 0.0025 |
|  | 21 dpi - 0 dpi | -0.2045187 | 0.201326 | 18 | -1.015856 | 0.3232 |

Notes: *: siginificant at level 0.05; “7 dpi – 0 dpi” means the percentage CD4^+^, CD8+ and CD4^+^/CD8^+^ of DPL and DLY pigs at 7 dpi compared with that at 0 dpi, respectively. “14 dpi – 0 dpi and 21 dpi – 0 dpi” are the same as above.
