# Supplementary material for: Genome-Wide Gene Expression Profiles in Lung Tissues of Pig Breeds Differing in Resistance to Porcine Reproductive and Respiratory Syndrome Virus
Source: PLoS One. 2014 Jan 23;9(1):e86101. doi: 10.1371/journal.pone.0086101 (PMC3900479; doi:10.1371/journal.pone.0086101)
Supplement: Table S2 — Statistical analysis for Cytokines and IgG. (DOC) [file pone.0086101.s003.doc]

**Table S2** Statistical analysis for Cytokines and IgG

| Item | Comparison | Difference | Standard error | Degrees of freedom | *t*-value | *p*-value* |
| --- | --- | --- | --- | --- | --- | --- |
| IL-1β | DPL - DLY | 4.38444 | 4.402448 | 18 | 0.995911 | 0.3325 |
|  | 7 dpi - 0 dpi | 53.02100 | 4.285028 | 34 | 12.373549 | 0.0000 |
|  | 14 dpi - 0 dpi | 4.03500 | 4.285028 | 34 | 0.941651 | 0.3530 |
| IL-2 | DPL - DLY | 5.91467 | 5.621793 | 18 | 1.052096 | 0.3067 |
|  | 7 dpi - 0 dpi | 61.14400 | 5.471851 | 34 | 11.174280 | 0.0000 |
|  | 14 dpi - 0 dpi | 5.45200 | 5.471851 | 34 | 0.996372 | 0.3261 |
| IL-10 | DPL - DLY | -26.98433 | 7.160869 | 18 | -3.76830 | 0.0014 |
|  | 7 dpi - 0 dpi | 72.13300 | 6.969878 | 34 | 10.34925 | 0.0000 |
|  | 14 dpi - 0 dpi | -22.85500 | 6.969878 | 34 | -3.27911 | 0.0024 |
| TNF-α | DPL - DLY | -7.96878 | 2.108352 | 18 | -3.77962 | 0.0014 |
|  | 7 dpi - 0 dpi | 21.24900 | 2.052119 | 34 | 10.35466 | 0.0000 |
|  | 14 dpi - 0 dpi | -6.73100 | 2.052119 | 34 | -3.28002 | 0.0024 |
| IFN-γ | DPL - DLY | 17.70944 | 4.685023 | 18 | 3.78001 | 0.0014 |
|  | 7 dpi - 0 dpi | -47.21400 | 4.560067 | 34 | -10.35380 | 0.0000 |
|  | 14 dpi - 0 dpi | 14.95900 | 4.560067 | 34 | 3.28043 | 0.0024 |
| IgG | DPL - DLY | -159.1686 | 92.17306 | 18 | -1.726845 | 0.1013 |
|  | 7 dpi - 0 dpi | 39.5404 | 86.27042 | 36 | 0.458331 | 0.6495 |
|  | 14 dpi - 0 dpi | 520.2063 | 83.52973 | 36 | 6.227798 | 0.0000 |

Notes: *: siginificant at level 0.05; “7 dpi – 0 dpi” means the percentage IL-1β, IL-2, IL-10, TNF-αIFN-γ and IgG of DPL and DLY pigs at 7 dpi compared with that at 0 dpi, respectively. 14 dpi – 0 dpi dpi are the same as above.
